# Lymphoid enhancer-binding factor 1, a representative of vertebrate-specific Lef1/Tcf1 sub-family, is a Wnt-beta-catenin pathway target gene in human endothelial cells which regulates matrix metalloproteinase-2 expression and promotes endothelial cell invasion

**DOI:** 10.1186/2045-824X-3-28

**Published:** 2011-12-14

**Authors:** Marina Planutiene, Kestutis Planutis, Randall F Holcombe

**Affiliations:** 1 The Tisch Cancer Institute at The Mount Sinai Medical Center, One Gustave L Levy Place, New York, NY 10029-6500, USA

**Keywords:** angiogenesis, β-catenin, cancer, endothelial cells, invasion assay, Lef1, MMP2, siRNA, Wnt signaling pathway

## Abstract

**Background:**

Wnt signaling is activated in many types of cancer and normal physiological processes. Various Wnt-related secreted factors may influence angiogenesis both in the tumor microenvironment and in normal tissues by direct action on endothelial cells. The mechanism of this Wnt action in angiogenesis is not well defined. We hypothesize that endothelial cells are responsive to Wnt signals and that Lef1, a member of the vertebrate-specific Wnt/beta-catenin throughput-inducing transcription factors' sub-family Lef1/Tcf1, mediates this responsiveness and promotes endothelial cell invasion.

**Methods:**

A human endothelial cell line, EAhy926 was exposed to Wnt3a or directly transfected with Lef1. Readouts included assessment of nuclear beta-catenin, Wnt throughput with a SuperTOPflash reporter assay, induction of Lef1 transcription, induction of matrix metalloproteinase (MMP)-2 transcription, cell proliferation and cell invasion through a matrix *in vitro*. The effects on MMP2 were also evaluated in the presence of Lef1 silencing siRNA.

**Results:**

Wnt3a increased nuclear beta-catenin and up-regulated Wnt/beta-catenin throughput. Wnt3a increased Lef1 transcription and activity of the Lef1 promoter. Both Wnt3a treatment and Lef1 overexpression induced MMP2 transcription but this effect was completely abrogated in the presence of Lef1 siRNA. Inhibition of Lef1 also reduced basal MMP2 levels suggesting that Lef1 regulates MMP2 expression even in the absence of exogenous Wnt pathway activation. Lef1 slightly increased proliferation of EAhy926 cells and increased invasion by more than two-fold.

**Conclusions:**

EAhy926 cells activate canonical Wnt signaling in response to Wnt3a ligand. The Wnt target Lef1 specifically regulates MMP2 expression in these cells and promotes endothelial cell invasion. The EAhy926 cell line provides a convenient alternative to primary human umbilical vein endothelial cells (HUVEC) in the study of angiogenesis and the role of Wnt signaling on endothelial cell function.

## Background

Many types of cancer exhibit activated Wnt signaling which contributes to tumor cell proliferation and inhibits differentiation [1]. In addition, secretion of Wnt ligands by malignant cells contributes to dynamic processes in the tumor microenvironment. Wnt 2, Wnt3a, Wnt 5b, and Wnt 16 expression is up-regulated in prostate cancer [2]; Wnt2 and Wnt5a are over expressed in colon cancer [3]. Down regulation of the Wnt inhibitory protein sFRP3 in the stroma and activation of epithelial-to-mesenchymal-transition, a process influenced by Wnt signaling [4], is associated with a poor prognosis in breast cancer [5].

In breast cancer, high endothelial marker content in tumor surrounding stroma is also a predictor of poor prognosis [5] and endothelial cells in the tumor microenvironment may be influenced by Wnt signals. In squamous cell carcinoma of the lung, intratumoral Wnt5a gene expression correlates with tumor angiogenesis and VEGF-A expression [6]. The Wnt pathway is a critical mediator of endothelial function [7]. In the tumor microenvironment, endothelial cells express multiple components of Wnt signaling pathways such as Wnt ligands, LRP5 [8], frizzled (Fz) receptors and soluble Wnt inhibitors [9], [10], and can respond to exogenous Wnt ligands [11]. The Wnt pathway is basally activated in subconfluent endothelial cells [9] and may promote endothelial cell proliferation. Wnt1 stabilizes active β-catenin and promotes both proliferation and formation of capillary-like networks in Matrigel [12], [10], an effect that may be mediated through induction of interleukin 8, a known angiogenic factor which is a direct target gene of Wnt/β-catenin signaling [13], [14] or hepatocyte growth factor [15]. However, others [16] have suggested that Wnt1 may have inhibitory activity on endothelial cell proliferation. A different Wnt ligand, Wnt3a, has been shown to induce endothelial cell proliferation and migration in the context of DVL3 phosphorylation [17].

Endostatin inhibits endothelial cells by inducing G1 arrest. This effect is mediated by inhibition of cyclin D1 transcription. Cyclin D1 is regulated by Lef1, a member of the Lef/Tcf transcriptional regulator family, which mediates β-catenin dependent (i.e., canonical Wnt pathway) transcription [18]. This suggests that the Wnt pathway is a target for endostatin, and that inhibition of Wnt signaling may be one mechanism by which endostatin is antiangiogenic. Soluble frizzled-related proteins, inhibitors of Wnt signaling, also inhibit vascular endothelial cell proliferation by delaying G1 and entry into S-phase [19].

Vertebrate vascular system is closed and contains more robustly streaming liquid compared with its analogue in invertebrates such as Hydra or *C. elegans*. Although Wnt pathway is involved in development of the vertebrate vascular system, the mechanisms of this involvement are largely unknown. Dietmar Gradl's lab recently delineated Lef/Tcf factor's evolution. Many invertebrates have only one Tcf factor from Tcf3/Tcf4 sub-family. For example, pop-1 (*C. elegans' *Tcf) can substitute Xenopus' Tcf3; Hydra's Tcf can substitute Xenopus' Tcf4 [20] and Dietmar Gradl's personal communication, 2011. Tcf1/Lef1 sub-family is present only in vertebrates. Members of this sub-family generate stronger Wnt throughput and Xenopus' secondary axis induction per protein unit than members of Tcf3/Tcf4 sub-family [21]. Tcf1 is presented in adult mammalian tissues mostly in its truncated dominant negative form that lacks β-catenin binding domain and inhibits the Wnt signaling [22] The higher activity of the only activating member from Lef1/Tcf1 subfamily in mammalian tissues Lef1 could be at least partially explained by a unique capacity of Lef1 to serve as a nuclear-β-catenin retention factor [23]. Does formation of closed vertebrate vascular system require this stronger signaling from Lef1-representative of Tcf1/Lef1 sub-family? This question is the focus of our work on human endothelial cell line.

We hypothesized that endothelial cells would be responsive to canonical Wnt signals and, since Lef1 increases the *in vitro *invasiveness of cancer cells [24], [25], and this is in many ways a similar process to endothelial cell migration through the basement membrane, that these responses would be mediated by Lef1. We show here that Wnt3a and Lef1 promote Wnt signaling in and invasiveness of endothelial cells. The novel information about the role of Lef1 in angiogenesis improves our understanding as to how the Wnt pathway regulates blood vessel growth both in normal physiological conditions and in cancer.

## Methods

### Cell lines

The human endothelial-like immortalized cell line EaHy926, derived from the fusion of human umbilical vein endothelial cells (HUVEC) with the bronchial carcinoma cell line A549, and expressing an endothelial-like phenotype is a generous gift from Dr. CJ Edgell [26]. It is grown in DMEM with 4.5g/l glucose and 10% FBS. Parental L-cells and L-cells stably producing Wnt3a were obtained from ATCC (ATCC#CRL-2647, ATCC#CRL-2648).

### Plasmid constructs & cell transfection

Expression plasmids utilized for transfection included a β-catenin expression construct, a Lef1 expression construct (FL9B), a Firefly luciferase reporter driven by the Lef1 promoter (B5 in pGL2 vector) provided by Dr. Marian Waterman (University of California, Irvine), and a pCMV Script vector control (Stratagene, La Jolla, California). Plasmids for assay readouts included SuperTOPflash and as a control SuperFOPflash kindly provided by Dr. Randall Moon (University of Washington), and *Renilla *luciferase, pGL4.74 (Promega Corporation, Madison, WI, Cat. #E6921). Transfection of plasmids was performed using Lipofectamine 2000 (Invitrogen, Cat. #11668-019) and Opti-MEM I Reduced Serum Medium (Invitrogen, Cat. #11058-021), or BioT reagent (Bioland Scientific, Cat. #B01-01) using the manufacturers' protocols.

### Treatment with Wnt ligands

Exposure to Wnt3a was accomplished utilizing two methods: endothelial cells were co-cultured side-by-side with Wnt3a producing L-cells [27] or they were treated with Wnt3a conditioned media. Wnt3a conditioned medium (CM) and control CM were prepared using LWnt3A cell line (ATCC#CRL-2647), and the parental cell line (ATCC #CRL-2648) according to procedures perfected in the laboratory of Dr. Roel Nusse [28]; for a preparation protocol, see http://www.stanford.edu/group/nusselab/cgi-bin/wnt/. Cells were analyzed after 24 hours of Wnt3a treatment.

### Evaluation of β-catenin by immunofluorescence staining

Cells were grown on Lab-Tek II chamber glass slides (Nalge Nunc International). They were fixed with 4% para-formaldehyde and treated 5 min at room temperature with 1% SDS in Tris-buffered saline for antigen retrieval. Donkey serum was utilized as a blocking agent to reduce background staining. Slides were incubated with primary antibody for 60 minutes at 25°C. Anti-β-catenin staining was performed with mouse monoclonal antibodies obtained from Transduction Laboratories (San Diego, clone C19220) or with anti-β-catenin rabbit polyclonal antibodies (Sigma, St. Louis, MO, clone C2206). Incubation with the primary antibody was followed by incubation with biotinylated secondary antibody (final concentration 1µg/ml), and subsequently with biotinylated horseradish peroxidase-avidin complex (Santa Cruz biotechnology, Santa Cruz, CA). As a substrate for peroxidase we used TSA Fluorescein System (Tyramide Signal Amplification, PerkinElmer Life Sciences, Cat. # NEL701A). Slides were visualized using fluorescent confocal microscopy; not less than 100 cells were analyzed with image quantification software to determine the relative β-catenin fluorescence.

### Wnt throughput assays

Firefly luciferase (SuperTOPflash or SuperFOPflash) and Renilla luciferase, the latest utilized as a control for the luciferase-based assays, were measured according to the manufacturer's instructions (Dual-Glo Luciferase Assay System and Bright-Glo Luciferase Assay System, Promega, WI, USA).

### Quantitative real-time PCR assays

The Lef1 mRNA levels were measured using cDNA synthesis and quantitative real-time PCR (qRT-PCR). RNA was purified with TRIzol reagent (Invitrogen). The pellet was dissolved in 20µl of DEPC-treated water. 1µg of the RNA was DNase treated, using Deoxyribonuclease I, amplification grade (Invitrogen, catalog No. 18068-015) according to the manufacturer's procedure. qRT-PCR was performed using One Step Applied Biosystems Sybr Green kit (Cat. #4310179) under the following conditions: reverse transcription reaction performed at 48ºC for 30 min followed by heating at 95ºC for 10 min. The PCR stage included 50 cycles of 15 sec dissociation at 95ºC and 1 min incubation at 60ºC. The identity of PCR products was confirmed by analysis of dissociation curves after the PCR. Actin was used for normalization. Primers used for the full length Lef1 mRNA were: Forward - CCGAAGAGGAAGGCGATTTAGCT, Reverse - GCTCCTGAGAGGTTTGTGCTTGTCT.

The MMP2 mRNA levels were measured by quantitative real-time PCR (qRT-PCR) utilizing the same parameters described above for Lef1. Primer sequences were [29]: Forward - CGCAGTGACGGAAAGATGTGGT, Reverse - AGAGCTCCTGAATGCCCTTGATGT

### Lef1 promoter activity

The B5 reporter construct, in which the LEF-1 promoter drives Firefly luciferase, was utilized to evaluate Lef1 promoter activity. Renilla luciferase was utilized as a control. Luciferase activity was measured using kits from Promega, Dual-Glo Luciferase Assay System (Cat. #E2920) and Bright-Glo Luciferase Assay System (Cat. #E2610), according to the manufacturer's instructions.

### Proliferation assay

Cell proliferation was determined by MTT assay using a Boehringer Mannheim Biochemica kit or by TACS^® ^XTT Cell Proliferation Assay kit from R & D Systems, (Cat. #4891-025-K) according to the manufacturer's recommendations. The cells were growing 96 - 120 hours.

### Invasion assay

Invasion of the Matrigel™ (BD Biosciences, Cat. # 354234) was performed according to the manufacturer's procedure using BD control inserts (BD catalog No. 354578) filled with a diluted Matrigel matrix. The Matrigel concentration was optimized as described by Albini [30]. 5 × 10^4 ^EAhy926 cells were plated over 3.3% Matrigel for one insert in a 24-well culture plate. Control inserts not coated with Matrigel were used for normalization. Cells were fixed, stained and counted at 72 hours after seeding.

### Gene silencing

Small inhibitory RNA (siRNA) targeting Lef1 β-catenin binding domain (CCCGAAGAGGAAGGCGATTTA and AGGGCGATCCTCAGAAGGAAA), and control random siRNA were obtained from Qiagen (Cat. #1027415). Transfection with siRNA was performed using BioT reagent (Bioland Scientific, Cat. # B01-01) following the manufacturer's protocol.

### Statistical considerations

Comparison of expression and activity levels for the various assays examined were made with an unpaired t-test, with the level of statistical significance defined as p < 0.05.

## Results and discussion

### Wnt3a induces canonical Wnt signaling in endothelial cells

**Figure 1:**
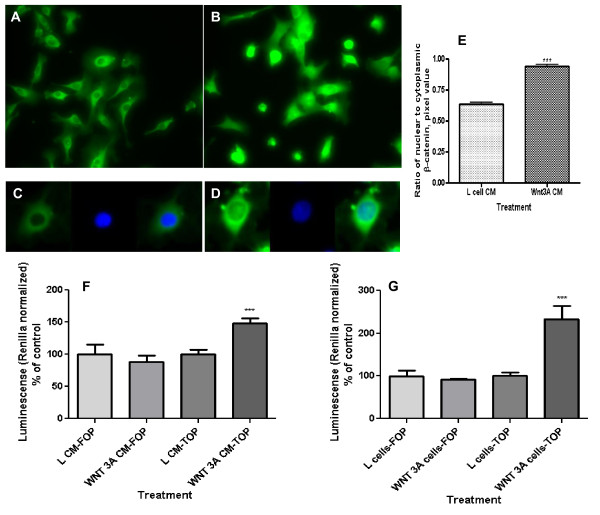
**Wnt pathway activation by extracellular Wnt3a as reflected in nuclear β-catenin content and level of Wnt throughput in EAhy926 endothelial cells**. (A) Control cells were not treated with Wnt. (B) Cell treatment with Wnt3a increased their cytoplasmic and nuclear β-catenin content. Cells were analyzed by immunofluorescence with primary antibodies against β-catenin, biotinylated secondary antibody, and subsequently TSA Fluorescein System. (C) A typical control cell with β-catenin staining (green fluorescence) and nuclear staining (blue fluorescence, DAPI). (D) A typical Wnt3a-treated cell. After treatment of cells with Wnt3a conditioned media (CM) β-catenin staining co-localized with nuclei. (E) Ratio of nuclear to cytoplasmic β-catenin in the EAhy926 cells. Wnt3A CM increased the nuclear/cytoplasmic ratio (**p < 0.0001). (F) Wnt throughput was measured by the SuperTOPflash reporter construct in EAhy926 cells. Wnt3a CM increased Wnt throughput (**p = 0.0038). No activation of the same reporter with mutated Lef/Tcf binding site (SuperFOPflash) indicated specificity of the Wnt3a-dependent cell response. (G) Wnt throughput as measured by the SuperTOPflash reporter construct in EAhy926 cells. Exposure to Wnt3a by side-by-side co-culture also increased Wnt throughput (**p = 0.0034). The SuperFOPflash reporter was used to check specificity of the response.

Primary human umbilical vein endothelial cells (HUVEC) respond to Wnt3a treatment [17]. To define the responsiveness of the EAhy926 cell line to Wnt signals, these cells were exposed to Wnt3a by conditioned media (CM) and also cultured in side-by-side co-culture with Wnt3a producing L-cells. Wnt3a CM promoted localization of β-catenin to the nucleus, a hallmark of canonical Wnt pathway activation (Figures 1A-D). The ratio of nuclear to cytoplasmic β-catenin increased significantly (Figure 1E; p < 0.0001). The basal level of Wnt signaling in EAhy926 cells, as measured by SuperTOPflash reporter activity, is low. Exposure to Wnt3a CM led to a significant 50% increase in Wnt pathway throughput (Figure 1F; p < 0.01). Incubation of EAhy926 in side-by-side co-culture with Wnt3a producing L-cells resulted in an even more dramatic 2.5-fold increase in reporter activity (Figure 1G; p < 0.01). SuperFOPflash reporter with mutated Lef/Tcf binding site does not react to Wnt3a. These data demonstrate existence of an intact canonical Wnt signaling pathway in EAhy926 cells responsive to exogenous Wnt3a; it is consistent with the response of HUVECs to exogenous Wnt signals which has been described by others [31], [32], [10], [17].

### Wnt3a increases Lef1 expression in endothelial cells

**Figure 2:**
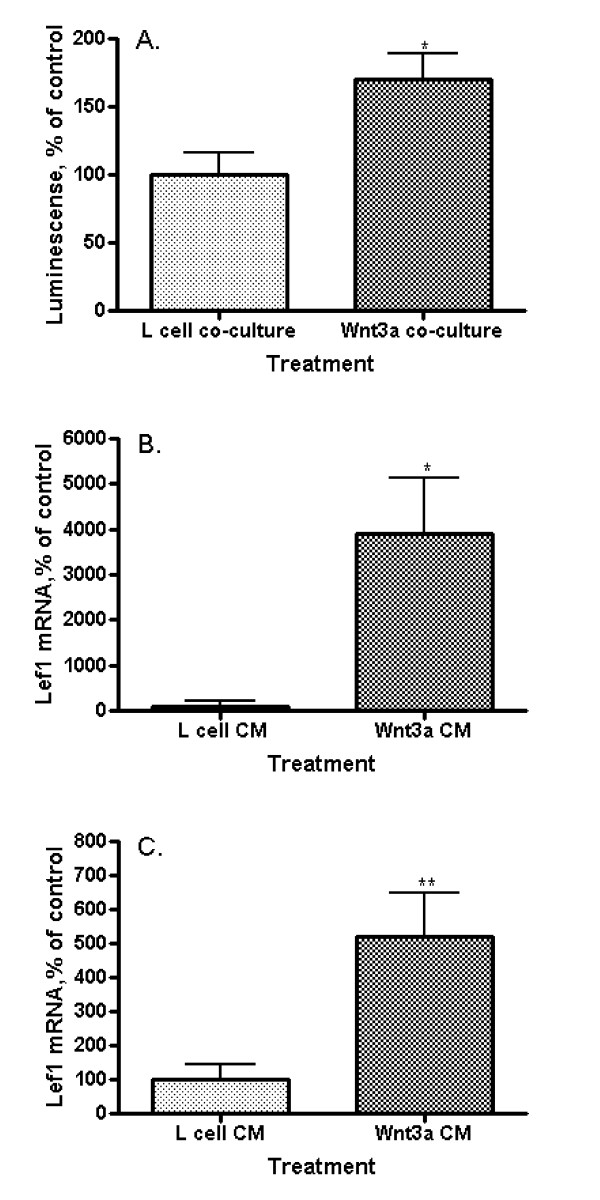
**Wnt3a effect on Lef1 transcription in endothelial cells**. (A) Lef1 promoter (B5) activity in EAhy926 cells. Exposure to Wnt3a in side-by-side co-culture activates the Lef1 promoter (*p = 0.0178). (B) Lef1 mRNA levels by quantitative real-time polymerase chain reaction (qRT-PCR) in EAhy926 cells. Wnt3a CM increases the amount of Lef1 mRNA (*p = 0.0379). (C) Lef1 mRNA levels by qRT-PCR in EAhy926 cells overexpressing β-catenin. Wnt3a CM increases the amount of Lef1 mRNA in cells with already activated by β-catenin Wnt signaling (**p = 0.0086).

Lef1 promoter activity in EAhy926 endothelial cells was measured by the response of a Lef1 promoter-driven reporter construct (B5) to Wnt3a in side-by-side co-culture with Wnt3a-producing L-cells. Lef1 promoter activity increased significantly when compared to co-culture with non-Wnt3a-producing parental L-cells (Figure 2A; p = 0.0178). Quantitative real time PCR, utilizing primers to detect full length (activating) Lef1 was performed on EAhy926 cells following exposure to Wnt3a. Wnt3a CM increased Lef1 mRNA expression 4000% (Figure 2B; p = 0.0379) though initial concentrations of Lef1 mRNA in unstimulated cells were very low. Lef1 mRNA levels were increased in EAhy926 cells transfected with β-catenin and responsiveness to Wnt3a was maintained with a 5-fold increase in mRNA levels following exposure to Wnt3a CM (Figure 2C; p = 0.0086). We define Lef1 to be a Wnt target gene in EAhy926 cells, since Wnt3a increases Lef1 message levels and promotes transcription driven off the Lef1 promoter. Lef1 is described as a canonical Wnt pathway target gene in other cell lines [33], perhaps regulated by E-tail containing Tcf transcription factors [34].

### Both Wnt3a and Lef1 elevate MMP2 mRNA level in endothelial cells

**Figure 3:**
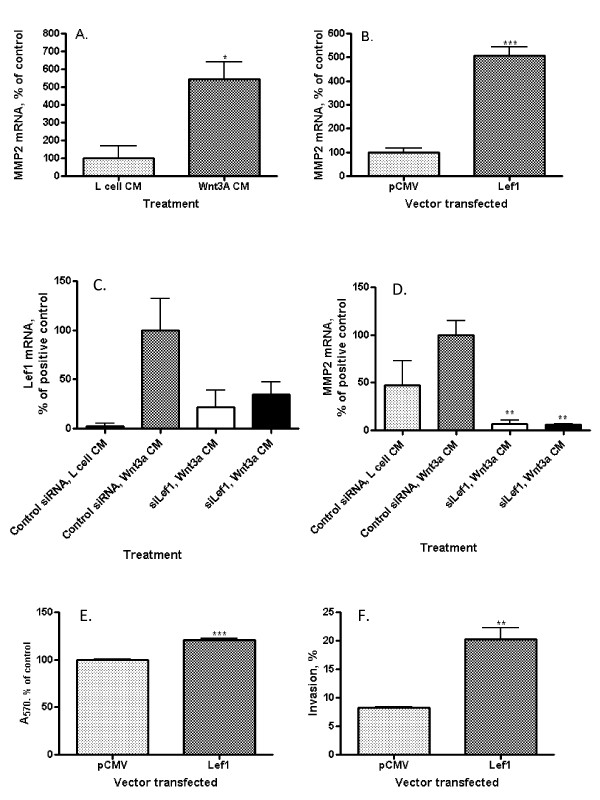
**Relationship between Wnt3a, Lef1, MMP2 mRNA, endothelial cell invasion, and proliferation**. (A) MMP2 mRNA levels by qRT-PCR in EAhy926 cells. Wnt3a CM increases the amount of MMP2 mRNA (*p = 0.0187). (B) MMP2 mRNA levels by qRT-PCR in EAhy926 cells. Transfection with a full-length Lef1 expression construct increases the amount of MMP2 mRNA (***p = 0.0005). (C) Lef1 mRNA levels by qRT-PCR in EAhy926 cells with control CM (column 1), following Wnt3a CM (columns 2, 3, 4) and in the presence of two different Lef1 siRNA (columns 3 and 4). The amount of Lef1 mRNA is reduced in cells treated with siRNA. (D) MMP2 mRNA levels by qRT-PCR in EAhy926 cells with control CM (column 1), following Wnt3a CM (columns 2, 3, 4) and in the presence of two Lef1 siRNAs (columns 3 and 4). Lef1 siRNA decreases MMP2 mRNA in EAhy926 cells from baseline and abrogates Wnt3a-mediated MMP2 induction (*p = 0.03). (E) Proliferation by MTT assay of EAhy926 cells. Transfection with a full-length Lef1 expression construct slightly increases proliferation after 96 hours (***p < 0.0001). (F) Invasion through Matrigel matrix by EAhy926 cells. Transfection with a full-length Lef1 expression construct significantly increases % invasion (**p = 0.0036).

Matrix metalloproteinase (MMP)-1 expression does not change in HUVECs following stimulation by Wnt3a [17] though MMP2 has been shown to respond to Wnt/β-catenin pathway stimulation in other cell models [29], [4]. MMP2 plays a key role in the invasion through extracellular matrix [31]. Exposure of EAhy926 cells to Wnt3a CM significantly increased MMP2 expression approximately 5-fold compared to control (Figure 3A; p = 0.019). Similarly, direct transfection of EAhy926 cells with a Lef1 expression construct resulted in a 5-fold increase in MMP2 mRNA levels (Figure 3B; p < 0.001) compared to vector-only control transfection. Both Wnt3a and Lef1 promote expression of MMP2 in EAhy926 cells. MMP2 hydrolyzes type IV collagen and other connective tissue substrates. During angiogenesis, the basement membrane is degraded, often by metalloproteinases, to facilitate invasion of endothelial cells through the membrane [35]. In some cancers, the level of MMP2 is increased; for example, in cases of human multiple myeloma MMP2 of bone marrow plasma cells is up-regulated [31]. MMP2 is a target of canonical Wnt signaling in T-lymphocytes [29] and in prostate cancer cells [4].

### siRNA-mediated inhibition of Lef1 reduces endothelial cell MMP2 mRNA levels and abrogates Wnt3a-dependent induction of MMP2 expression

Lef1 is one of a family of transcription factors which, in association with β-catenin, regulate gene expression as a terminal event in the Wnt signaling pathway. In order to determine whether Lef1 specifically regulates MMP2 expression, Wnt3a CM-induced increases in MMP2 mRNA expression were evaluated under normal conditions (control siRNA) and after the knockdown of Lef1 (siLef1). Cellular Lef1 mRNA levels were reduced by 60-75% by two different Lef1 siRNAs (Figure 3C). The amount of Lef1 mRNA is reduced in cells treated with Lef1siRNA even without Wnt3a treatment; however without stimulation Lef1 mRNA is hardly detectable (Additional file 1).

These siRNAs completely abrogated the Wnt3a CM-induced increase in MMP2 expression (Figure 3D; p = 0.03). Additionally, Lef1 siRNAs reduced MMP2 mRNA below basal levels despite exposure to Wnt3a. This implies that in EAhy926 both basal and Wnt-augmented expression of MMP2 is dependent specifically upon Lef1 and that endogenous MMP-2 transcription is tightly regulated by Lef1, even in the absence of Wnt pathway activation. It seems that Lef1 specifically regulates MMP2 expression since transfection with a full-length Lef1 expression construct does not affect significantly the amount of MMP1 mRNA (Additional file 2).

### Lef1 increases endothelial cell proliferation and invasion

Wnt signals are proliferative for most cell types. In EAhy926, transfection with Lef1 slightly increased cell proliferation as measured by an MTT assay (Figure 3E; p < 0.0001). Longer growth time confirms the result (Additional file 3). More importantly, consistent with its effect on MMP2 expression seen here and prior studies in breast cancer models [24], Lef1 promoted EAhy926 cellular invasion through a Matrigel matrix, increasing this 2.5-fold from 8.23 ± 0.17% to 20.27 ± 1.96% (Figure 3F; p = 0.0036). Our data supports the hypothesis that Lef1 is an essential component of the regulation of proliferation and invasion of EAHy926 endothelial cells.

Lef1 also directly affects invasion of EAhy926 endothelial cells through a matrix *in vitro*, likely, at least in part, through its actions on MMP2. *In vivo*, this property is analogous to endothelial cell migration through the extracellular matrix. Anti-Lef1 therapy has been postulated to reduce invasion of cancer cells [24], [25]. Both Lef1 and MMP2 might also be considered as potential new targets for anti-angiogenic therapy.

While our data points to the role of canonical (β-catenin) Wnt signaling in angiogenesis regulation, additional evidence suggests that non-canonical pathways may also play a role. Wnt5a, a Wnt ligand typically inhibitory for canonical signal transduction, can promote endothelial cell proliferation and survival via a non-canonical ERK 1/2 dependent process [36]. The non-canonical planar cell polarity component DAAM1 has also been implicated [37]. Even traditional canonical Wnt ligands such as Wnt3a may also affect endothelial cell function via non-canonical processes involving Cam Kinase II [17]. Further research defining the roles and interactions of canonical and non-canonical Wnt signaling pathways will improve our understanding of the complex regulation of endothelial cells.

Since Lef1-dependent Wnt throughput is stronger per protein unit than Tcf4-dependent signaling prevalent for example in a normal mammalian gut, we might need to develop Lef1-specific inhibitors for the angiogenesis control [38].

Our studies were performed in an immortalized cell line derived from endothelial cells, and thus may not be completely analogous to *in vivo *conditions. However, when we compare our data concerning the endothelial cell line EAhy926 with the results obtained by others from study of primary HUVECs [17], it is apparent that these two cell systems have many similarities (Table 1). At least for the study of Wnt/β-catenin signaling and its role in angiogenesis, EAhy926 is a readily available alternative to HUVECs.

**Table 1:** Comparison Wnt-mediated regulation of the EAhy926 immortalized cell line and HUVECs.

**Human endothelial immortalized cell line EAhy926, treated with Wnt3a **(our results)	**Primary HUVECs, treated with Wnt3a **[17]
Increase of nuclear β-catenin	Stabilization of total cellular β-catenin

Activation of Wnt/β-catenin throughput	Induction of DVL3 phosphorylation

Up-regulation of full length Lef1	Data are not available

Increase in proliferation (MTT) after Lef1 transfection	Increase in proliferation (BrdU incorporation)

Elevation of MMP2	No change in MMP-1

Lef1 siRNA induced inhibition of MMP2 response	Data are not available

Increase of invasion	Increase of migration

Data are not available	No effect on survival

## Conclusions

In conclusion, our data, and recent findings of others, demonstrate that canonical (β-catenin) Wnt signaling is important for endothelial cell function in angiogenesis. We additionally define Lef1 as the key downstream effector for this pathway in endothelial cells through regulation of MMP2 expression and cell invasion.

## List of abbreviations

Fz : frizzled receptor; HUVEC : human umbilical vein endothelial cells; Lef1 : Lymphoid enhancer-binding factor 1; LRP5 : low-density lipoprotein receptor-related protein 5; MMP2 : metalloproteinase-2; Tcf1 : T-cell factor 1; TSA : Tyramide Signal Amplification.

## Competing interests

The authors declare that they have no competing interests.

## Authors' contributions

RFH conceived the study, had oversight over experimental design and execution and contributed to manuscript preparation. MP and KP contributed equally, performing cell based assays, molecular studies, and analyzing the data. Each contributed to manuscript preparation. All authors have reviewed and approved the final manuscript.

## Supplementary Material

Additional file 1 **Effect of Lef1 siRNA on Lef1 mRNA in the absence of Wnt3a**. Full length Lef1 mRNA levels by qRT-PCR in EAhy926 cells treated by control CM (without Wnt3a) in the presence of control RNA (column 1) and Lef1 siRNA (columns 3 and 4). The amount of Lef1 mRNA is reduced in cells treated with siRNA even without stimulation by Wnt3a (**p = 0.0018). Click here for file

Additional file 2 **Effect of Lef1 expression on MMP1 mRNA**. MMP1 mRNA levels measured by qRT-PCR in EAhy926 cells. Transfection with a full-length Lef1 expression construct does not affect significantly the amount of MMP1 mRNA. Click here for file

Additional file 3 **Prolonged proliferation of EAhy926 cells measured by XTT assay**. Transfection with a full-length Lef1 expression construct slightly increases proliferation of EAhy926 cells after 120 hours (**p = 0.0077). Here we used the TACS^® ^XTT Cell Proliferation Assay from R & D Systems. Click here for file
