# Supplementary figures and images for: Lymphoid enhancer-binding factor 1, a representative of vertebrate-specific Lef1/Tcf1 sub-family, is a Wnt-beta-catenin pathway target gene in human endothelial cells which regulates matrix metalloproteinase-2 expression and promotes endothelial cell invasion
Source: Vasc Cell. 2011 Dec 14;3:28. doi: 10.1186/2045-824X-3-28 (PMC3269378; doi:10.1186/2045-824X-3-28)

## Slide 1
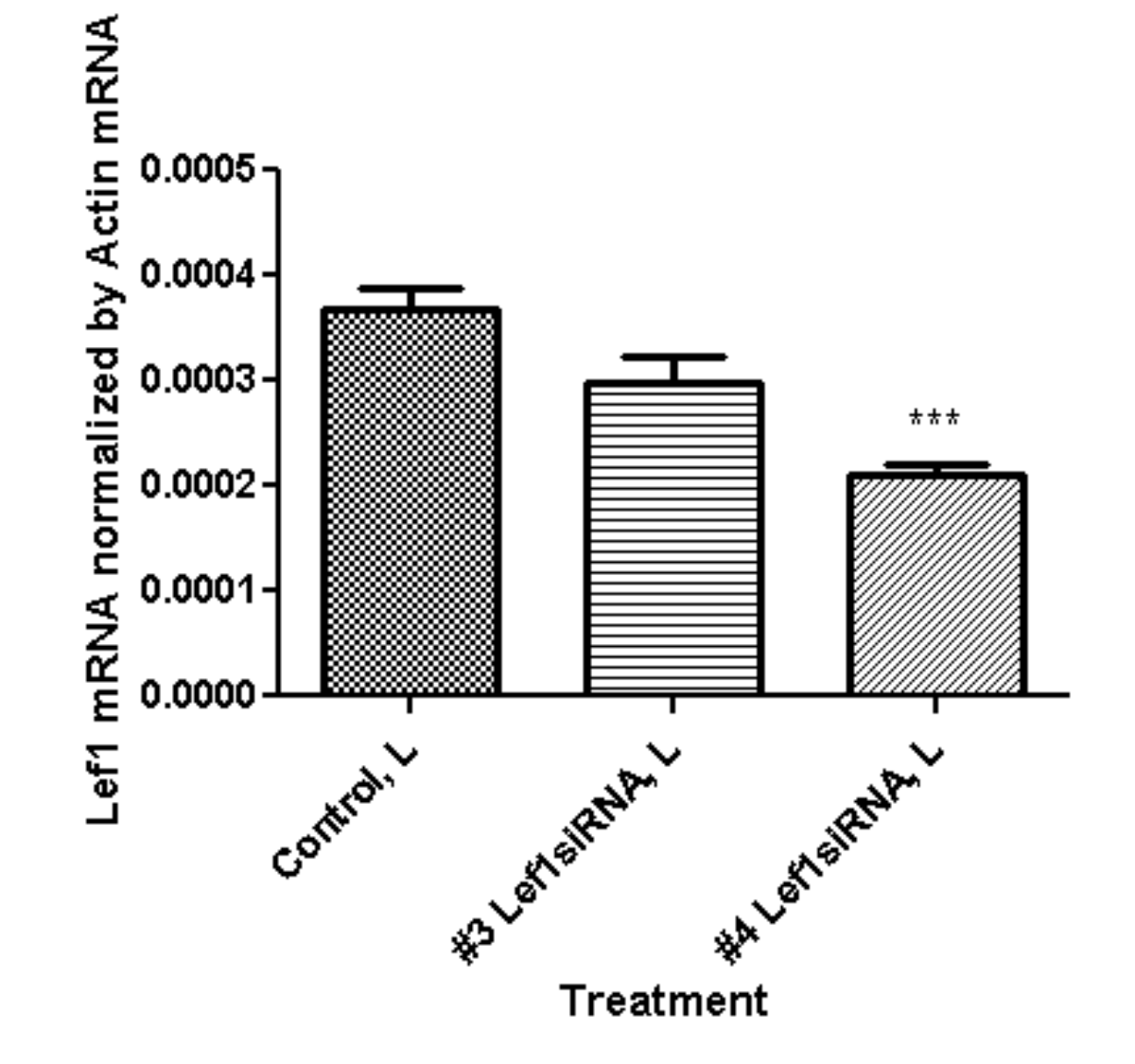

Supplement: Additional file 1 — Effect of Lef1 siRNA on Lef1 mRNA in the absence of Wnt3a. Full length Lef1 mRNA levels by qRT-PCR in EAhy926 cells treated by control CM (without Wnt3a) in the presence of control RNA (column 1) and Lef1 siRNA (columns 3 and 4). The amount of Lef1 mRNA is reduced in cells treated with siRNA even without stimulation by Wnt3a (**p = 0.0018). [file 2045-824X-3-28-S1.PPTX]

## Slide 1
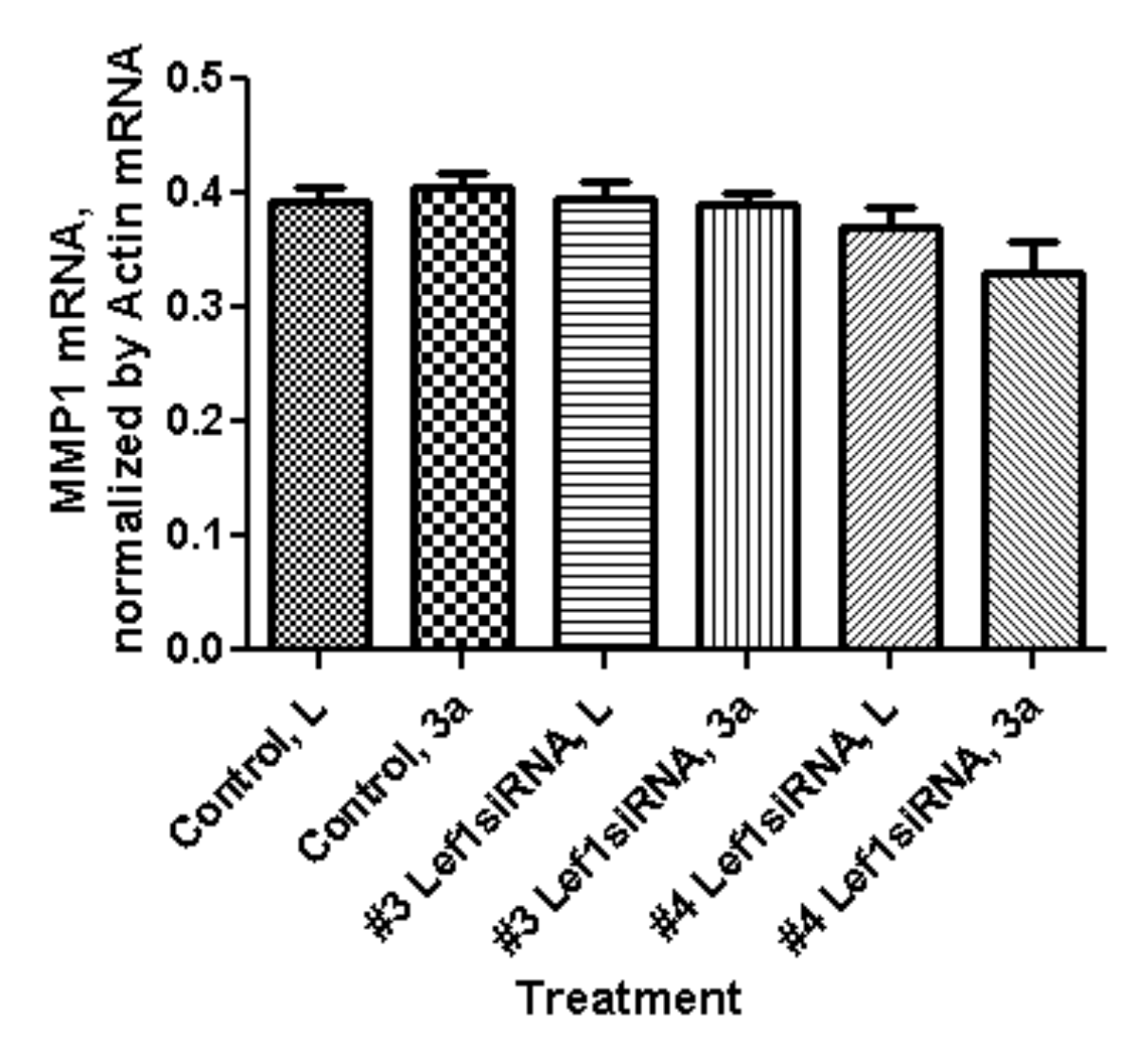

Supplement: Additional file 2 — Effect of Lef1 expression on MMP1 mRNA. MMP1 mRNA levels measured by qRT-PCR in EAhy926 cells. Transfection with a full-length Lef1 expression construct does not affect significantly the amount of MMP1 mRNA. [file 2045-824X-3-28-S2.PPTX]

## Slide 1
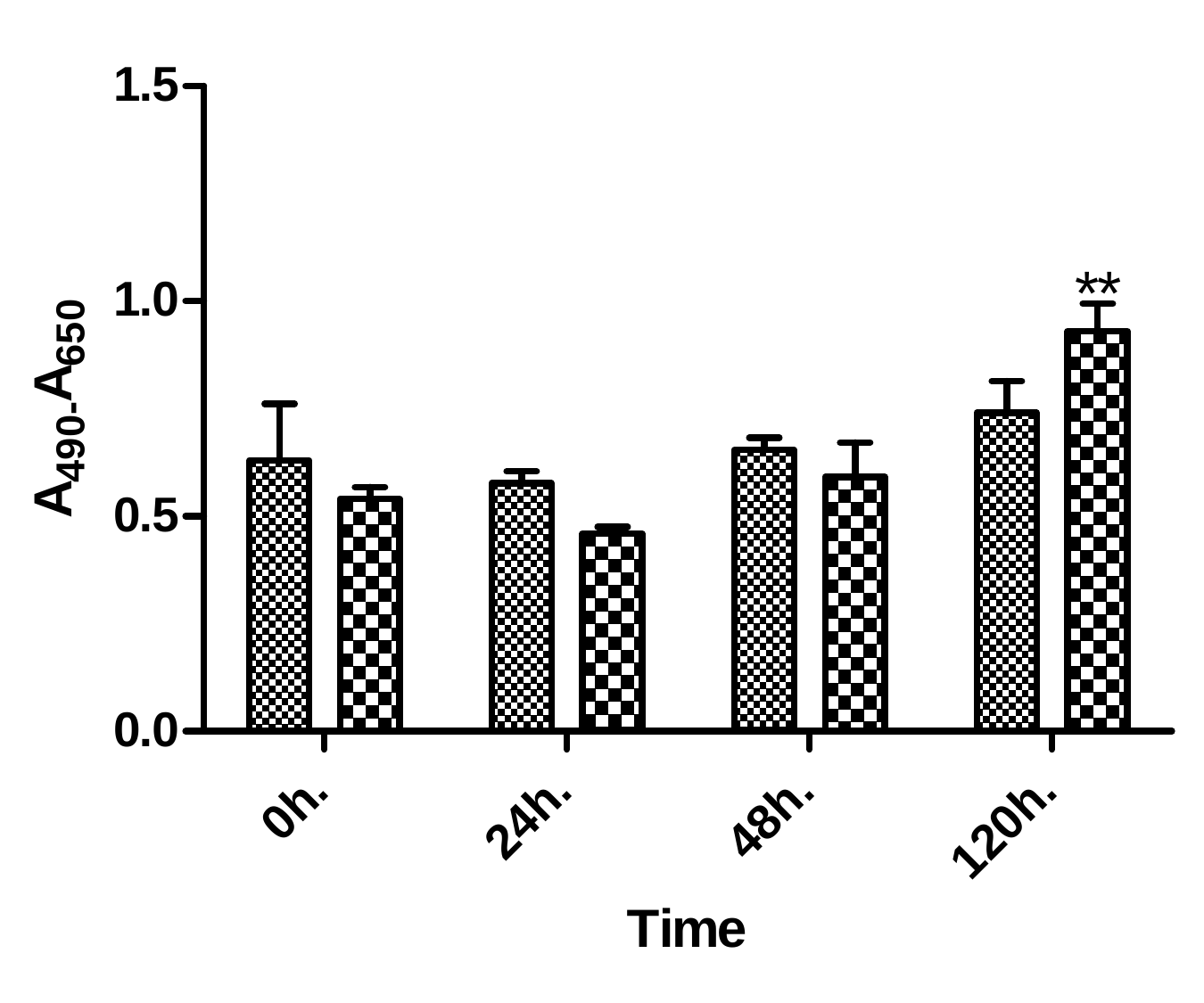

Supplement: Additional file 3 — Prolonged proliferation of EAhy926 cells measured by XTT assay. Transfection with a full-length Lef1 expression construct slightly increases proliferation of EAhy926 cells after 120 hours (**p = 0.0077). Here we used the TACS® XTT Cell Proliferation Assay from R & D Systems. [file 2045-824X-3-28-S3.PPTX]
